# AR DriveSim: An Immersive Driving Simulator for Augmented Reality Head-Up Display Research

**DOI:** 10.3389/frobt.2019.00098

**Published:** 2019-10-23

**Authors:** Joseph L. Gabbard, Missie Smith, Kyle Tanous, Hyungil Kim, Bryan Jonas

**Affiliations:** ^1^Grado Department of Industrial and Systems Engineering, Virginia Tech, Blacksburg, VA, United States; ^2^Industrial and Systems Engineering, Oakland University, Rochester, NY, United States; ^3^Virginia Tech Transportation Institute, Virginia Tech, Blacksburg, VA, United States; ^4^Department of Mathematical Sciences, United States Military Academy, West Point, NY, United States

**Keywords:** augmented reality, head-up display, conformal graphics, driving simulator, human machine interface

## Abstract

Optical see-through automotive head-up displays (HUDs) are a form of augmented reality (AR) that is quickly gaining penetration into the consumer market. Despite increasing adoption, demand, and competition among manufacturers to deliver higher quality HUDs with increased fields of view, little work has been done to understand how best to design and assess AR HUD user interfaces, and how to quantify their effects on driver behavior, performance, and ultimately safety. This paper reports on a novel, low-cost, immersive driving simulator created using a myriad of custom hardware and software technologies specifically to examine basic and applied research questions related to AR HUDs usage when driving. We describe our experiences developing simulator hardware and software and detail a user study that examines driver performance, visual attention, and preferences using two AR navigation interfaces. Results suggest that conformal AR graphics may not be inherently better than other HUD interfaces. We include lessons learned from our simulator development experiences, results of the user study and conclude with limitations and future work.

## Introduction

While once the provenance of select academic and government labs, augmented reality (AR) has now been applied in many contexts and delivered over a myriad of hardware technologies. Successes have been documented regarding, for example, smartphone AR on the go (DüNser et al., 2012; Shea et al., 2017), tablet based AR in classrooms (Bower et al., 2014), spatial AR in architecture (Tonn et al., 2008), and head-worn AR in military and medical applications (Shen et al., 2013; Gans et al., 2015). However, handheld AR notwithstanding, it is quite possible that the largest AR user base will soon be automobile drivers using see-through automotive head-up displays (HUDs) to view both screen-positioned 2D and conformal 3D AR content.

Indeed, recently we have seen renewed interest using HUDs in driving, due in part to the commercialization of next-generation AR technologies. Automobile manufacturers are beginning to field AR HUD technologies (86 models in the US offered HUDs in 2018), with marketing teams pushing for more advanced AR HUD user interfaces. By 2020, HIS Automotive predicts there will be 9.1 million HUDs sold.

Moreover, in the very near future, we expect increasingly large AR HUD field of views, affording placement of information in many locations; from windshield-fixed positions to conformal graphics that are perceptually attached to real-world referents. In the same timeframe, we expect an increase in semi-autonomous vehicles where drivers *must still attend* to both the road scene and system information (likely provided via AR HUDs), creating the perfect storm for potentially dangerous and distracting AR HUD interfaces.

While next-generation AR HUDs will provide a fundamentally new driving experience, we currently do not know how to effectively design and evaluate user interfaces (UIs) in this space. With new AR HUDs capable of rendering images over large areas at varying depths, the visual and cognitive separation between graphical and real-world visual stimuli will be increasingly more difficult to quantify. As we move toward widespread use of next-gen AR HUDs in transportation, we need to better understand how to manage UI designs that are not simply *atop the environment*, but instead are *an integrated part of the environment*.

Without new research capabilities, HUD UI researchers and practitioners are left to base HUD UI design and assessment on current (and dated) understanding of traditional in-vehicle information systems. Common in-vehicle display assessment methods were developed based on data collected in vehicles in the early 2000s (Administration NHTS, 2013), and recent research suggests these assessment methods have limited applicability to AR HUDs (Smith et al., 2016). Thus, as we start fielding, and designing for, new AR HUD displays, we must also develop our understanding of AR HUD effects on visual attention and driver performance. In a design space that affords fundamentally different user experiences, we must pose the question: “*How do AR HUD user interfaces that are necessarily visually integrated into a highly dynamic primary task space affect driver performance?*” Driving simulators provide a method of rapidly iterating on AR HUD design in realistic driving scenarios without the danger or cost of on-road testing.

To this end, this paper reports our experiences creating a relatively low-cost, full-scale driving simulator designed to examine AR HUD usage effect on driver performance and behavior. The remainder of the paper describes the hardware and software technical implementation in detail, followed by a user study to demonstrate the utility of the driving simulator and concludes by presenting lessons learned from our multi-year endeavor creating and testing an AR HUD driving simulator.

## Related Work

To explore opportunities of driving simulation for AR user interface design and evaluation, we briefly examine human-subject studies that incorporated a various range of (1) simulator hardware, (2) optical see-through AR displays, and (3) software to realize conformal graphics for driver-vehicle interfaces. For more information about driving simulation in general (e.g., current state-of-art technology, applications, capabilities, and limitations), see a comprehensive handbook (Fisher et al., 2011).

Regarding fidelity of driving simulation (i.e., visual stimuli, vehicle control, and motion), a wide range of driving simulator hardware has been used in empirical studies on AR applications depending upon the research questions addressed. The lowest fidelity settings are often a combination of desktop computers, monitors and game controllers (Neurauter, 2005; Kim and Dey, 2009; Weinberg et al., 2011; Charissis et al., 2013; Kim et al., 2013; Tran et al., 2013; Politis et al., 2014; Sharfi and Shinar, 2014; Tippey et al., 2014). For example, Sharfi and Shinar (2014) prototyped an AR visibility enhancement system for nighttime driving that highlights lane markers using a desktop computer, DEXXA game controllers, and a 126 × 60 cm monitor and found that augmented road edges have positive effects on drivers' confidence and workload while reducing their ability to detect unexpected obstacles. Other researchers have used medium fidelity driving simulators that typically consist of a fixed-based real car cab with wall projection screens (Tonnis and Klinker, 2006; Caird et al., 2008; Plavšic et al., 2009; Olaverri-Monreal et al., 2012; Saffarian et al., 2013; Schall et al., 2013; Wai-Tat et al., 2013; Bolton et al., 2015). Fu et al. conducted a user study in a driving simulator with a GM Saturn real-car cab on a fixed base (Wai-Tat et al., 2013). The user study showed that the proposed AR forward collision warning improved driving performance but induced risky driving behavior especially among young drivers. A few user studies have been conducted in a high-fidelity driving simulator with motion-based real car cabs with wide field of view projection screens, in-vehicle displays for mirrors and center console displays (Medenica et al., 2011; Lorenz et al., 2014). For example, Medenica et al. (2011) evaluated the usability of three navigation aids in a high-fidelity real-car cab atop a motion-base which is able to simulate vehicle motion for braking and accelerating. The user study showed benefits of a conformal AR navigation aid showing a virtual route hovering above the road against traditional map-view or street view navigation aids presented on a center console display. Lastly, SILAB (WIVW, 2019), a commercially available driving simulator, supports a flexible, wide range of simulation fidelity from desktop systems with gaming control inputs to multi-channel projected scenes with real vehicles placed on motion platforms. Similar to our work presented herein, SILAB supports physiological measurement, video capture of driver and passengers from arbitrary angles, eye tracking, real-time connection protocols (such as TCP/IP, UDP, and CAN bus), and support for secondary task integration. From materials available online, it is not clear if separate AR HUD hardware has been successfully integrated into SILAB. However, it is certainly plausible that the infrastructure as described would support such an endeavor.

For AR displays, most researchers have simulated AR HUDs by presenting AR graphics directly within driving scene (with no physical AR display; Caird et al., 2008; Kim and Dey, 2009; Plavšic et al., 2009; Charissis and Papanastasiou, 2010; Medenica et al., 2011; Dijksterhuis et al., 2012; Olaverri-Monreal et al., 2012; Kim et al., 2013, 2016; Saffarian et al., 2013; Schall et al., 2013; Wai-Tat et al., 2013; Lorenz et al., 2014; Politis et al., 2014; Sharfi and Shinar, 2014), while some installed in-house prototypes (Tonnis and Klinker, 2006; Langlois, 2013; Tran et al., 2013), aftermarket c, or head-worn displays inside driving simulators (Sawyer et al., 2014; Tippey et al., 2017). Generally speaking, from our experience, integrating graphics directly into the driving scene (via computer graphics or video) does not afford the same accommodative and/or cognitive switching (Gabbard et al., 2019) that a separate AR display does; an important component for research that wishes to faithfully examine the effects of AR HUDs on driver's visual attention. Moreover, home-made AR HUDs (e.g., using tablets and semi-transparent combiners) may suffer from ghosting and other visual artifact that can impact user study results unless extreme care is put into its construction.

Kim et al. (2013) simulated an aftermarket HUD by presenting a virtual hardware form factor of the HUD (24 × 8° field of view) with semi-transparent AR forward collision warning and blind spot warning via the virtual display. Schall et al. (2013) simulated a full windshield HUD for AR collision warning by directly highlighting road hazards with virtual boxes integrated into the driving scene. Tonnis and Klinker (2006) prototyped an in-house HUDs by using a combiner and a small projection screen for AR graphics separate from a large wall projection screen for driving scene. Similarly, Lauber and Butz (2013) simulated a head-up display using an LCD display and combiner mirror with 70% transparency, to compare screen-fixed presentation of speed, speed limit, collision warning, and basic navigation information to head-worn AR presentation via a Vuzix StarTM 1200 HM. Pfannmueller et al. (2015) used a mock-up of a contact analog head-up display (cHUD) to present AR graphics atop a video projection of driving scenes to explore various AR navigation display concepts. While details are lacking, the cHUD appears similar to others that use a tablet or monitor reflected via semi-transparent combiner (as opposed to commercial head-up or head-worn AR display). While this system provides a quick method for assessing AR HMI design concepts, it does not support manual driving, nor does it appear to easily support investigation of conformal AR graphics.

A nice on-road study by Wiesner et al. (2017) uses a commercial prototype head-up display in a real vehicle to understand driver performance with AR interface designs in real driving environments. The work integrated a high-precision Global Navigation Satellite System (GNSS) and an advanced driver-assistance systems (ADAS) horizon system to study the effectiveness of “AR-like” visualizations of imminent intersections, highway exits and roundabouts. The authors do not conformally integrate AR graphics into the scene in part because the graphics represent a future event; thus tight visual integration is not explicitly warranted. The authors employ eye tracking and results regarding the effect of AR on drivers' glance behavior are similar to our results presented herein: namely that an AR HUD-based presentation helps drivers keep their eyes in the direction of road longer, with shorter glances toward the instrument cluster, and slightly longer mean glances toward the HUD (as compared to a head-down display).

Conformal graphics in driving simulators have been realized mostly by direct integration of AR graphics into computer-generated driving scene without separate displays (Caird et al., 2008; Kim and Dey, 2009; Plavšic et al., 2009; Charissis and Papanastasiou, 2010; Medenica et al., 2011; Kim et al., 2013; Schall et al., 2013; Wai-Tat et al., 2013; Lorenz et al., 2014; Politis et al., 2014; Sharfi and Shinar, 2014). The few instances found in literature that present conformal AR graphics use Wizard of Oz (Bolton et al., 2015), computer-vision-based object detection (Wu et al., 2009), and communication between driving simulation software and AR application (Tran et al., 2013). Lorenz et al. (2014) prototyped AR warnings for restricted lanes due to emergency situations by presenting green safe path or red dangerous path by integrating conformal graphics into the driving scene using the same rendering pipeline as the driving environment. Bolton et al. (2015) presented drivers with a seemingly autonomous driving scenario including pre-recorded navigation arrows visible through an optical see-through HUD which correspond with a specific driving scenario that were manually-triggered by researchers. Wu et al. (2009) played driving footage in front of a driving simulator and overlaid AR bounding boxes through the windshield to highlight detected road signs by computer-vision technology. Finally, Tran et al. developed a capability of presenting real-time conformal graphics via communication with driving simulation software that transmitted information about road geometry, other road actors and traffic signals. They presented AR graphics to visualize predicted path of oncoming traffic for left turn aid. However, details about the system configuration and software architecture were not reported (Tran et al., 2013).

Conducting AR studies with conformal AR graphics on-road is very difficult due to the difficulty in tracking the driver's position and orientation. At first thought, this may seem like an easy proposition given today's real-time kinematics-enabled GPS, accelerometers, vision-based, and LIDAR sensing capabilities. However, the small vibrations and undulations caused by the road surface, tires, and vehicle suspension actually makes tightly registering conformal graphics to the driving scene difficult. Exacerbating the problem is the fact that any slight variations in graphics' movements (relative to the real-world referents) are easily detected by the human visual system effectively rendering the potential benefits of conformal graphics disconcerting, distracting or confusing. Until such ability to “lock” conformal AR graphics into the near and far driving scene is established, the use of driving simulators designed specifically to study the effects of AR graphics on driver performance and behavior, such as our system presented herein, is critically needed. It should be noted that while these technical tracking capabilities advance, we can still examine, both in simulation and on-road, AR graphic designs that are more forgiving of tracking and pose estimation errors. For example, Wiesner and Klinker (2017) present an AR visualization for navigation which aimed to overcome errors in current GPS turn by relying solely on distance to next turn. Interestingly, their results suggest that precision of a conformal graphic may not be the sole determinant of effectiveness, since their “sails” AR design that required lower precision via conventional GNSS system was preferred over a high-precisions conformal arrow design.

In sum, when reviewing the literature, it is clear that several researchers have and continue to engage in meaningful AR HUD research using a myriad of hardware, software, and experimental methodologies. From our review, and to the best of our knowledge, our AR DriveSim is unique in that it provides several synergistic capabilities including: fully immersive vehicle cab and projection system, physiological capture, and measurement (e.g., of eye and driver behavior), custom electronics for communications and control of vehicle, synchronization of data streams associated with vehicle dynamics, AR interfaces and driver/passengers, autonomous driving, force-feedback to the steering wheel, integrated after-market head-up display, and the ability to render full color, animated, conformal AR graphics.

## Driving Simulator for AR Interface Research

**Figure 1:**
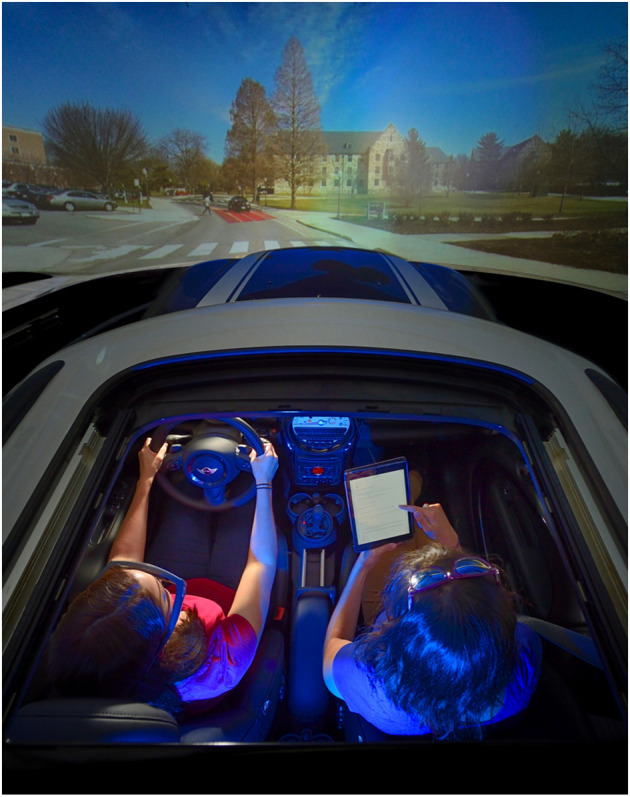
A bird's eye view of the Mini Cooper half cab with participant and experimenter. While the work presented herein focuses on CG-based driving capabilities, the testbed also supports alternative forms of driving studies (e.g., video-based).

In this section, we report the details of a multi-year effort to build an AR driving simulator (hereafter referred to as *AR DriveSim*). From the onset, we established several guiding principles. (1) Embed an actual vehicle cab into a wide field of view 3D projection space (Figure 1). That is, we wished to create a high degree of immersion as described by Witmer and Singer, that is a “psychological state characterized by perceiving oneself to be enveloped by, included in, and interacting with an environment that provides a continuous stream of stimuli and experiences,” and well as a high degree of place illusion (Witmer and Singer, 1998; Skarbez et al., 2017). (2) Employ an actual HUD to display AR (and other) graphics, and not simply project or integrate “simulated AR graphics” into the driving scene. (3) Embrace flexibility in the testbed design to afford many different types of human-subjects studies with a focus on AR HUD usage. (4) Empower researchers to collect a suite of dependent measures to characterize human performance and behavior including driver performance metrics, visual attention, and gaze patterns, objective measures of mental workload, and video-based measures of head, hand and feet movements. The following sections describe key components of our AR DriveSim in hopes that these contributions help others develop similar capabilities.

### AR DriveSim Hardware

**Figure 2:**
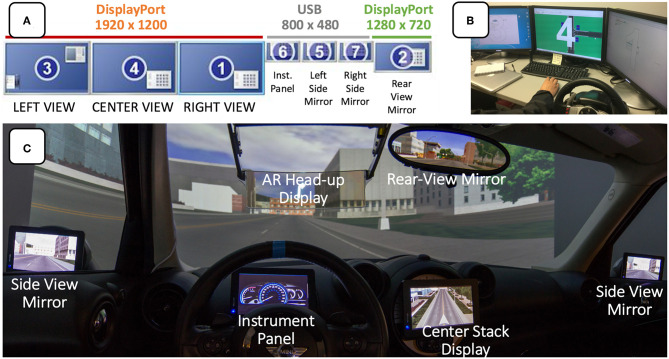
The logical arrangements of 7 AR DriveSim screens with resolution and physical connections noted **(A)**; experimenter's control station **(B)**, and an annotated view from inside the Mini Cooper cab **(C)**. Note that the AR HUD and Center Stack displays are connected to a separate computer dedicated to UI presentation.

At its core, the AR DriveSim is a projection-based, monoscopic virtual environment, whereby users “locomote” the environment as a driver of an automobile. In our system, the VR content is provided via MiniSim, a 3D driving simulator software developed at the University of Iowa's National Advanced Driving Simulator research center. MiniSim 2.2 executes on a desktop computer with an Intel Core i7 processing running @ 3.70 GHz, with 64 gigabytes of DDR4 RAM running Windows 10. The driving scene is rendered by a PNY NVIDIA Quadro P4000 graphics card and projected via DisplayPort @ 1,920 × 1,200 using three (warped and blended) Epson Powerlite Pro G6900WU NL projectors. In this hardware configuration, MiniSim provides smooth rendering of up to about 1 million triangles at 60 frames per second. We route these three main forward views through Tripp Lite hardware to mirror the viewports onto three desktop monitors (Figure 2B) to provide an experimenter's view and control station.

For the projection surface, we mounted a professional grade lace-and-grommet screen by Draper that is 93″ high by 360″ long, onto a custom U-shaped, curved frame (73″ inch radius). The projection screen uses a Contrast Gray XH800E smooth gray viewing surface that provides enhanced color contrast and black levels, and is especially useful for our application that uses three projectors with high lumen output. The frame consists of 1½ inch rolled aluminum tubing at both the top and bottom, with 1 × 1 square aluminum tubing structural uprights spaced approximately every 2′.

The centerpiece of our driving simulator is the front half of a 2014 Mini Cooper automobile. The vehicle was donated from a major car insurance company that kindly removed the engine and transmission prior to delivery. Once delivered, we tested the electrical components and then completely disassembled the vehicle, including all trim, seats, airbags, dash components, and more until just the frame remained. The back half of the cab was removed and discarded, and the top half of the remaining cab was temporarily removed. The two cab halves were relocated into a lab, where the back-end of the bottom half was mounted on a frame with casters (the front-end of bottom half supported by original tires). The top half was the reattached and we then reassembled all the previously removed components (from supporting sub-structures to finished trim pieces) and tested the reassembled vehicle electrical systems.

We then incorporated additional displays to support side view mirrors, rear view mirrors, digital instrument panel, and flexible center-stack displays. Specifically, we added three Lilliput 7-inch USB LCD video monitors (800 × 480) connected via powered USB hubs and DisplayLink software to serve as side view mirrors and customizable digital instrument panel (Figure 2). We placed an ASUS PB328Q 32″ widescreen LCD monitor behind the cab (and rendered content accordingly) to afford natural use of the optical rear-view mirror. The rear-view monitor is connected via DisplayPort at 1,280 × 720 to optimize performance in the three main forward projected views. To increase place illusion, we added a consumer grade subwoofer and speakers in the engine compartment to render real-time audio such as engine noise.

Lastly, we added a suite of additional equipment to assist in capturing participant behavior. A set of three Axis P1204 3.7 mm mini HD covert pinhole network cameras were placed (1) on the rear-view mirror (facing the participants face), (2) in the driver footwell (capturing foot behavior such as hovering over brake pedal), and (3) on the center of the cab ceiling pointing at participants' hands on the steering wheel. The cameras are connected to the NOLDUS Observational Suite, which affords synchronized video across the three IP cameras as well as with a direct digital video feed of the driving scene from the drivsim computer. The AR DriveSim also contains both SMI ETG 60 Hz and Tobii Pro Glasses 2 100 Hz wireless eye tracking glasses with forward looking scene camera that allows us to carefully assess drivers' gaze allocation; an especially critical capability for understanding how AR HUD interface designs affect drivers' visual attention. We capture physiological measures of driver workload using a Mio LINK heart rate monitor to capture heart rate variability (Meshkati, 1988), and RedScientific's Detection Response Task to provide an objective measure of residual attentional capacity using the dual-task paradigm (Sala et al., 1995).

### Simulator Controls and Interface System

While there are many ways to connect physical cab controls to simulation software, we chose to decode the Mini Cooper's exiting Controller Area Network (CAN) bus so that we could leverage existing high-speed control data streams. A *CAN bus* is a serial data communication protocol developed by the BOSCH Corporation to mitigate the challenges associated with data transfer and exchange among a vehicle's controllers, sensors, instruments, and other electrical components (Ran et al., 2010). By leveraging bi-directional CAN bus communication, it is possible to, for example, read steering wheel position, pedal positions, and button presses, and also manipulate the speedometer, tachometer and other elements from simulation in real-time. While there are many online resources describing the principles of the CAN bus architecture and wide array of application areas, manufacturer-specific CAN bus IDs are much more difficult to locate as they are generally not released to the public. Since we were unable to find CAN bus IDs for a 2014 Mini Cooper, we used a combination of off-the-shelf on-board diagnostics scanning tools, an Arduino CAN bus Shield, an oscilloscope and professional grade automotive diagnostic computers to reverse engineer the set of CAN bus IDs, variable length payloads, and values for critical Mini Cooper functions.

**Figure 3:**
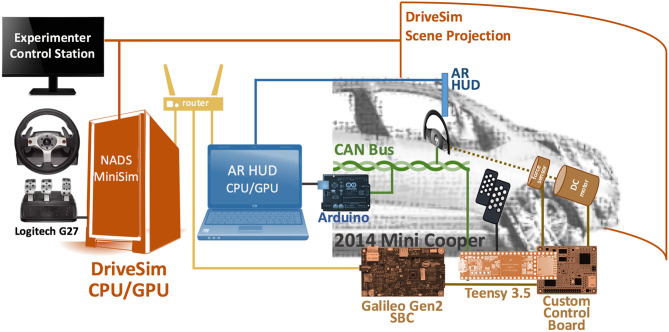
The major computing components of our AR DriveSim communicate via UDP (yellow). A set of off-the-shelf and custom microcontrollers (brown) pass driving control inputs read from CAN bus (green) and other sensors to the AR DriveSim computer (orange). A control board (brown) further manages a DC motor to provide force feedback to the steering wheel. A separate computer (blue) renders 3D graphics on an AR HUD by synchronizing its virtual camera position in real-time with the AR DriveSim computer. A set of experimenter controls (black) assist in coordinating experiments.

To facilitate communication between the Mini Cooper and MiniSim software, we integrated a single board computer (SBC), microcontroller and custom control board to collect and send CAN bus messages, analog voltages from several custom-installed linear potentiometers, and a few OEM sensors (Figure 3).

The microcontroller is used to manage the low-complexity, highly-repetitive tasks such as receiving CAN bus messages and reading the analog voltages from the various sensors. We used a Teensy 3.5 because of the built-in CAN bus receiver function, a high number of digital and analog general-purpose input/output, and the flexibility of several protocols for communicating with other systems. The more complex functions of the interface system are managed by a Linux-based SBC that receives parameters from the microcontroller, formats and scales them as needed, and finally composes and sends the data as UDP packets across wired Ethernet to the MiniSim computer. We initially utilized an Arduino Yún as the SBC but following a serial communications issue, we switched to an Intel Galileo Gen2.

The Teensy uses the CAN bus interface to access control data such as steering wheel position data, button presses, etc. We installed three linear potentiometers to measure the position of the accelerator pedal, brake pedal, and automatic gearshift position. Each of these parameters is linearly scaled to single byte-sized values and transferred over a serial connection between the Teensy and the Galileo. Upon starting, a python control script stored on the Galileo begins a handshake exchange with the Teensy to establish common timing for the communication scheme. Once communication is started between the two devices, the SBC determines the timing of the transmissions by transmitting a single byte to the Teensy. In response, the Teensy transmits all the steering and position values it has received from the sensors and CAN bus via a two-wire serial connection at 115,200 bps. Once received, the Galileo linearly rescales these values per the MiniSim specifications and packages them into a UDP packet. Testing indicates that this custom interface system reliably transmits 100 packets per second. Although we have not formally measured the end-to-end latency, we expect it to be minimal given (1) MiniSim parses incoming UDP data at 60 Hz, and (2) our own empirical observation.

The Teensy communicates with the Cooper's CAN bus using the FlexCAN library (Pimentel and Fonseca, 2004) and a handler that extracts the required information at the time of reception of each CAN bus frame. Once we knew the frame ID of the required parameters and of the structure of these frames, it was very easy to harvest the needed information as it came across the bus. The linear potentiometers used to measure the position of the accelerator and brake pedals are connected to the pedals via plastic-sheathed control cables (we could not decode pedal position in CANBUS). The potentiometers are supplied with 3.3 V and are read at the Teensy's standard 13-bit resolution. The OEM spring-return of the pedals benefits our system by also returning the potentiometers to their “zero” position. As the actual range of mechanical movement of the pedals and potentiometers can be affected by friction and other factors; our analog reading routine updates the minimum and maximum read value for both pedals and utilizes these values to map the current reading to a value between 0 and 255 for transmission to the Galileo. Similar to pedal setup, a plastic-sheathed control cable connects the automatic gearshift to the linear potentiometer which is also supplied with 3.3 V. We used pre-measured values of the voltages associated with the various gears on the automatic transmission to determine the position transmission in the analog reading routine.

To increase the place illusion afforded by the driving simulator experience, we repurposed the electric power steering feature of the Mini Cooper to provide force feedback as well as return-to-center to the steering wheel as is experienced in a normal vehicle. To support these sensorimotor contingencies, we designed a opto-isolated MOSFET H-bridge circuit to allow a brushed DC motor that is coupled to the steering shaft to move the steering wheel as desired. By changing the pulse-width modulation duty cycle, we are able to change the force feedback intensity to vary with the simulated vehicle speed. This H-bridge circuit was built on a custom-designed and printed circuit board that we term the “control board.” The control board also contains the Teensy, power circuitry, CAN bus connection header, as well as the connections for the linear potentiometers and any future sensors and electronics.

Launching and stopping the python script on the Galileo is accomplished from a python-based graphical user interface (GUI) accessible on the MiniSim computer that utilizes a secure shell to issue commands to the Galileo. The control processes are run in the background of the Galileo to provide robustness in the event of a timeout of the secure shell session or other issue. By providing a simple GUI to the communication layer, all researchers regardless of computing background can easily launch and monitor communications between the Mini Cooper, its microcontrollers and simulation software.

We also added a Logitech G27 game-based racing wheel and pedals to not only assist in driving scenario development and testing, but more importantly, to allow for Wizard of Oz autonomous driving studies (e.g., how AR HUDs can assist handover between manual and autonomous driving). The aforementioned Python GUI allows researchers to switch between Mini Cooper controls (i.e., participant manually driving) and game controller (e.g., experimenter driving as an autonomous agent).

### AR Head-Up Display Implementation

#### AR HUD Hardware

To support our research on the effects of AR interfaces on driver performance and behavior, we integrated a Pioneer Carrozzeria Cyber Navi Head-up display. The Cyber Navi is an optical see-through, fixed focal length (~3 m) laser-based display designed to be mounted on the interior roof in place of a sun visor. We mounted the HUD on a rail along the interior roof of the Mini Cooper so that it can be positioned at varying distances (8–24 inches) from the driver's eyepoint. According to the manufacturer, the Cyber Navi supports a ~17° horizontal field of view, which is consistent with our experiences calibrating the HUD image to the MiniSim driving scene.

As a laser-based display, the Cyber Navi can produce bright images at 12,000 cd/m^2^ and has an ambient light sensor and automatic dimming capability. The automatic dimming however created color-rendering issues in our simulation environment; at low light levels (i.e., dark simulator room) the HUD not only dims but also has a strong color bias toward green. That is, white graphics appear green at low lighting levels. To remedy this, we mounted a single LED on a potentiometer directly in front the HUD light sensor. When the LED is lit, the HUD adjusts by creating brighter images resulting in good color rendering. We then applied 20% visible light transmission tinting to the lens to better match the luminance of the HUD graphics to the projected driving scene.

#### AR HUD Software

Generally speaking, the HUD can render a VGA video source from any VGA-compatible computer and software. This is convenient, as we have successfully conducted user studies using PowerPoint to render 2D screen-fixed text and symbols to assess driver distraction and visual attention with varying HUD positions and UI complexity (Smith et al., 2016, 2017). As shown in Figure 3 (in blue), our simulator contains an Arduino microcontroller and CAN-Shield that parses steering wheel button presses from the CAN bus and routes them to the AR HUD computer by emulating a USB connected keyboard. The Mini Coopers' steering wheel buttons are conveniently arranged to afford a left and right directional-pad (plus two additional buttons located on the right side of steering wheel). In this arrangement, researchers can quickly design experiments that present a series of visual stimuli and employ up to 10 different button presses to explore HUD interface issues such as menu navigation, manual conformation of UI selections, self-paced psychophysical studies, and more.

However, conformal AR HUD graphics require a more complicated software platform consisting of data traffic control, data transformation, and scene graph components. In our current system, we implement these components as MiniSim's UDP route table, a middleware Python script, and an X3D/JavaScript scene graph, respectively.

Data passes between components as UDP packets containing information as defined by MiniSim's route table–a customizable construct that allows us to specify which MiniSim variable are packaged and broadcast over the network at 60 Hz (as defined by the output rate of MiniSim). In order to present AR graphics, we transmit MiniSim's simulated vehicle position and orientation within the scene. This data is then used to continuously update the position and orientation of the X3D camera.

Depending on the nature of the data output by the traffic controller, it may need to be transformed to meet the specification of the scene graph component. To meet X3D's pose specifications, own-vehicle coordinates in MiniSim must be negated along the z-axis. MiniSim's yaw, pitch, and roll values are then used to generate a single rotation vector and magnitude. This transformed data is used to match the pose of X3D's viewpoint to that of the driver within the simulation. This means presentation of conformal AR HUD graphics is defined solely by X3D's viewpoint pose relative to MiniSim's scene.

Timing of AR HUD graphics' behavior is done through the use of MiniSim's road pad trigger events which, when driven over by participants, generate event specific network data traffic. For example, in the user study presented below, road pad triggers create data packets that inform the AR HUD software that the driver has encountered an augmented driving segment, and consequently begin rendering the desired AR HUD graphics. The data selected to inform the behavior of conformal graphics is adaptable as a callback mechanism to launch procedures defined in the AR HUD scene graph component. The MiniSim route table can also be configured to send position and orientation data on the nearest 20 dynamic scene objects (e.g., other vehicles, pedestrians, etc.). Such information can also be used to render real-time conformal graphics such visual pedestrian alerts and labels for nearby traffic.

For reference, it should be noted that for the study presented below, we were able to render conformal AR HUD graphics using X3D on a fairly small computer: Intel i5 2,400 s @ 2.5 ghz, 4 gigs ram, Ubuntu 14.04 LTS, running CPU graphics. More complicated AR HUD imagery, either in presentation or behavior, would be well-suited for newer computing and graphics hardware.

#### Calibrating the AR HUD

Because the physical HUD position may need to change to accommodate different driver height and seat positions, it is important that a calibration procedure be performed to ensure accurate perceptual registration of conformal graphics to the driving scene. To accomplish this, participants first sit in the driver's seat and position the seat to a comfortable position. We have participants perform a coarse positioning of the AR HUD combiner (which is hinged along the top edge) such that top and bottom edges of the combiner align with a prepared calibration image projected onto the curved screen. This ensures that the AR HUD is correctly positioned vertically in the scene so that it, for example, covers the roadway.

**Figure 4:**
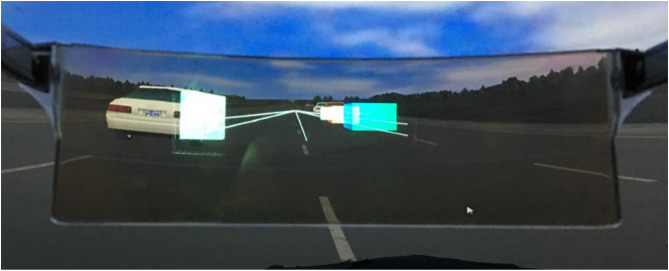
A participant's view as they calibrate the AR HUD 3D graphics by viewing carefully placed 3D shapes and perspective lines overlaid onto a static road scene with known geometry.

Next, participants check to ensure that conformal AR graphics perceptually appear in the correct location. For this step, we created a simple highway scenario containing a visible horizon and four vehicles parked at known positions along either side of the highway. The AR HUD software draws boxes around each car as defined by a shared absolute coordinate system. Additionally, the software draws lines correlating to the highway's lane markings to the point of convergence as viewed in simulation (Figure 4). By using incremental keyboard controls defined in the AR HUD software to manipulate field of view, aspect ratio, viewpoint pitch, and viewpoint position, we are able to quickly align these graphics with respect to their simulation counterparts. The calibration routine implicitly leverages each participant's tendency to align augmented and simulation graphics using their dominant eye, ensuring perceptually accurate augmentations of the driving scene.

## AR HUD User Study

### Purpose

After building and refining all driving simulator components, we performed a user study to demonstrate the testbed's research capability. We were especially interested in comparing traditional 2D HUD style graphics to conformal AR graphics since a majority of AR work aims to study the effect of conformal graphics on driver/operator performance.

Automotive manufacturers are already implementing 2D screen-fixed AR HUD graphics (i.e., graphics are displayed in a fixed position on the HUD screen) in vehicles on the road today. These screen-fixed images are used to display a variety of information, including navigation directions. One area garnering much interest with automotive manufacturers is the potential for georeferenced, world-relative graphics that might be “fixed” in a single location in the world, or dynamic, moving relative to the world, but appearing as part of the world. One of the most common use-cases for these world-relative graphics is navigation, as cues within the world can provide drivers with information to help them navigate throughout complex environments. These two types of graphic use the same technology to convey similar information (where to go) in very different ways. For this reason, our purpose with this study was to compare visual attention, driving behaviors, and experience when using two different types of AR HUD navigational graphics: screen-relative and world-relative, both fixed in location.

### Experimental Design

**Figure 5:**
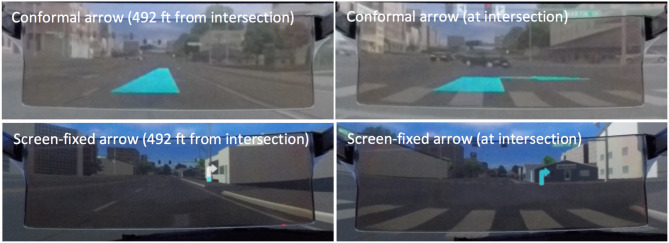
The user study examined two HUD display conditions: a *conformal* arrow integrated into road scene **(top)**, and, a *screen-fixed* arrow which filled as drivers approached turns **(bottom)**. For each, the initial state of the graphic appeared at 392 feet from the interaction (left panels), and disappeared after participants traversed intersections (right panels).

We compared two different navigation display conditions (Figure 5): a conformal arrow (Conformal) and a screen-fixed arrow (Screen-fixed). *Conformal* arrow was rendered on the HUD and appeared as if it was on the road and blue in color. As participants approached the turn, they “drove over” the arrow as if it was part of the road. Screen-fixed displayed turn directions using a 2D arrow rendered on the HUD, oriented left or right as appropriate, and inspired by current navigation systems. The vertical portion of the Screen-fixed arrow filled as participants approached the turn indicating the distance-to-turn.

### Methods

After study approval from Virginia Tech's Institutional Review Board, we recruited participants using flyers posted on campus, shared with faculty, and email listserv postings. In addition, several participants asked permission to share their experience participating with other friends in the area, and some people chose to participate based on these referrals. Interested individuals contacted the researchers via email to ask any questions regarding the study and to schedule the data collection session, if desired. Upon arrival in the lab, participants consented to participate and entered the driving simulator where they were fitted with eye tracking glasses and adjusted the seat to their comfort. They then performed a familiarization drive to get comfortable with driving simulator setting and vehicle dynamics. We instructed them to drive 30 mph and obey all traffic rules and norms including traffic signals. If they exceeded the speed limit by more than 10%, an audible siren sound was presented indicating that they needed to slow down. The familiarization drive lasted for a minimum of 5 min, until they indicated that they were comfortable with driving the simulator vehicle and the researchers also confirmed that they were able to maintain vehicle control while stopping, starting, turning, and driving straight. After the familiarization drive, we calibrated the HUD vertically and horizontally.

Participants experienced the navigation display conditions in a series of drives. Each drive took place in a large city and included eight turns: four right turns and four left turns, all of which were cued by the navigation system and lasted between 6 and 12 min. Differences in duration were largely due to individual differences and randomized traffic patterns. In addition, participants were instructed to attend to oncoming traffic and cross traffic while turning and driving throughout the city. Half of the turns (two left, two right) had cross traffic consisting of a platoon of eight vehicles.

Throughout the drive, glance behavior and gaze direction was captured via SensoMotoric Instruments (SMI) eye tracking glasses; which recorded audio, forward facing view, and gaze location for each participant. After data collection, we used SMI BeGaze 3.6.40 analysis software to analyze participants' fixation allocation using manually defined areas of interest. We used the Noldus Observation Suite to record video of the forward-looking road scene independent of participants' gaze direction. This video footage was used to identify participants' risk-taking behaviors. After each drive, participants completed a short series of questionnaires which included workload and usability measures.

We collected complete data for 22 participants, all of whom had a US driver's license for longer than 1 year (mean 4.6 years, maximum: 19 years, minimum: 2 years). Thirteen males (mean age 20.3 years) and nine females (mean age 20.4 years) participated. On average, participants drove 7,918 miles per year.

### Analysis and Results

We conducted a mixed effects linear model which allowed us to account for individual participant differences as a random effect as seen in de Bruin et al. (2008). We analyzed a series of dependent measures of workload and usability, glance behavior, driving behavior, and risk taking. For each dependent measure, we fit our model in JMP Pro 12 accounting for the effect of the independent measures (display type, order, participant gender, turn direction, presence of traffic) and second-order interactions of these effects. As this was an exploratory study, the scope of this paper only includes detailed discussion regarding the individual influence of display type on dependent measures.

#### Workload and Usability Measures

**Figure 6:**
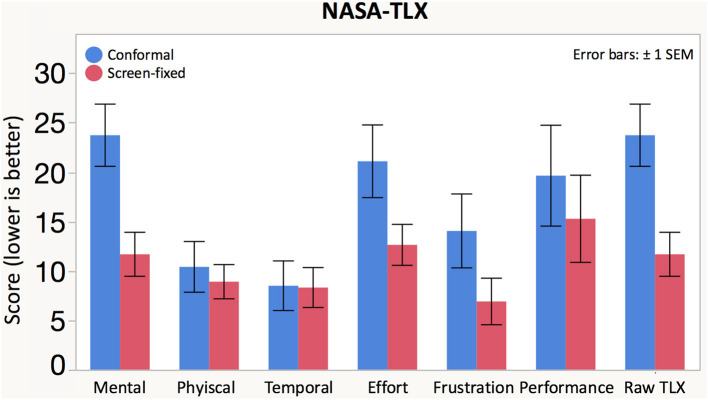
Participants rated the NASA-TLX sub-scores on a scale of 0 (low demand) to 100 (high demand). The average of the sub-scores comprised the Raw TLX score.

Participants self-reported workload using NASA-TLX (Hart and Staveland, 1988) after each drive. There was a significant effect of navigation display on mental demand, effort, and overall Raw TLX score (the average of all sub scores; see Figure 6, Table 1). The Screen-fixed display resulted in lower mental demand, effort, and overall workload than the Conformal display.

**Table 1:** Means, standard deviations (between parentheses), and *F-* and *p*-values for the repeated measures ANOVA.

**Dependent variable**	**Display type**		**Significance**	
	**Conformal**	**Screen-fixed**	***F***	***p***
**Workload measured with NASA TLX**				
Mental demand (%)	23.7 (14.8)	11.7 (10.4)	*F*_(1, 17.58)_ = 11.2505	0.0036^*^
Physical demand (%)	10.4 (12.0)	8.91 (8.12)	*F*_(1, 17.53)_ = 0.3030	0.5889
Temporal demand (%)	8.50 (11.8)	8.32 (9.48)	*F*_(1, 18.41)_ = 0.0001	0.9923
Effort (%)	21.1 (17.2)	12.6 (9.71)	*F*_(1, 18.57)_ = 5.1149	0.0359^*^
Frustration (%)	14.1 (17.5)	6.91 (11.0)	*F*_(1, 18.86)_ = 2.2575	0.1495
Performance (%)	19.6 (23.9)	15.3 (20.7)	*F*_(1, 17.52)_ = 3.0807	0.0967
Raw TLX (%)	16.2 (11.4)	10.6 (7.90)	*F*_(1, 18.02)_ = 6.6204	0.0191^*^
**Usability**				
Distraction (%)	16.0 (24.4)	5.05 (7.19)	*F*_(1, 16.48)_ = 4.4355	0.0509
Driving impact (%)	22.1 (29.4)	5.64 (9.39)	*F*_(1, 18.29)_ = 9.8564	0.0056^*^
Navigation (%)	22.9 (24.9)	4.91 (8.42)	*F*_(1, 18.16)_ = 15.3798	0.0010^*^
Trust (%)	9.23 (15.2)	2.00 (4.96)	*F*_(1, 19.15)_ = 4.8508	0.0401^*^
Viewing (%)	28.6 (31.2)	3.82 (7.19)	*F*_(1, 18.46)_ = 25.5842	0.0000^*^
**Glance behavior**				
Max HUD graphic glance duration (s)	3.33 (2.49)	1.17 (1.45)	*F*_(1, 24.95)_ = 33.526	0.0000^*^
Mean HUD graphic glance duration (s)	1.48 (1.40)	0.71 (1.05)	*F*_(1, 298.8)_ = 5.888	0.0158^*^
Glance count (#)	6.00 (3.31)	3.54 (2.39)	*F*_(1, 1351)_ = 4.2756	0.0389^*^
% Around HUD graphic (%)	28.6 (21.0)	41.5 (23.2)	*F*_(1, 24.32)_ = 16.257	0.0005^*^
% HUD graphic (%)	34.8 (19.3)	11.8 (13.1)	*F*_(1, 26.07)_ = 32.464	0.0000^*^
% Off-HUD hazards (%)	17.4 (13.2)	26.4 (21.6)	*F*_(1, 26.61)_ = 0.137	0.7142s
% On-HUD hazards (%)	17.4 (18.9	18.1 (20.0)	*F*_(1, 27.26)_ = 0.006	0.9377
% HUD (%)	80.9 (13.4)	71.5 (22.0)	*F*_(1, 28.03)_ = 0.014	0.9055
**Driving behavior**				
Mean lane position (ft)	0.37 (0.84)	0.41 (0.88)	*F*_(1, 319.5)_ = 0.3977	0.5287
St. Dev of vehicle speed (mph)	8.29 (3.39)	8.47 (3.24)	*F*_(1, 320.5)_ = 0.3842	0.5358
St. Dev. of steering degrees (°)	26.3 (18.0)	26.0 (18.3)	*F*_(1, 321.6)_ = 0.0506	0.8221
St. Dev. of lane position (ft)	1.07 (0.61)	1.04 (0.53)	*F*_(1, 319.8)_ = 0.5251	0.4692
Peak deceleration (ft/s^2^)	9.12 (5.31)	9.06 (5.16)	*F*_(1, 319.8)_ = 0.0124	0.9114
Stop distance (ft)	36.2 (17.3)	36.1 (17.2)	*F*_(1, 40.82)_ = 0.0576	0.8116
**Risk taking**				
Gap size (ft)	111 (31.9)	114 (31.8)	*F*_(1, 42.25)_ = 0.1559	0.0

Bold and

* *indicates statistical significance*.

**Figure 7:**
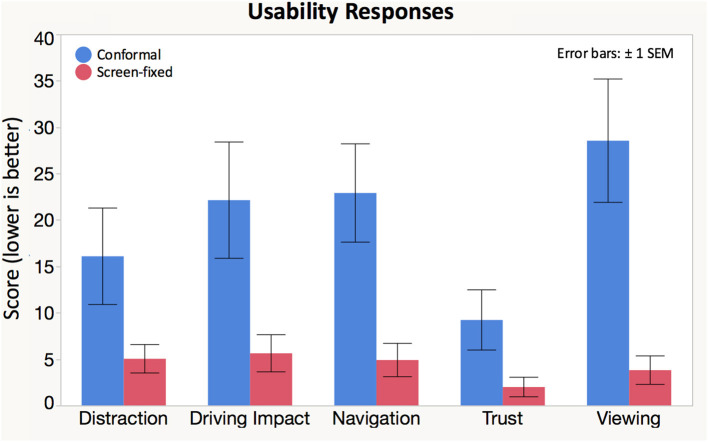
Participants rated the following statements on a scale of 0 (strongly agree) to 100 (strongly disagree): (1) “I did not find this interface distracting.” (2) “Using this interface had a positive impact on my driving.” (3) “It was easy to navigate while using this interface.” (4) “I trusted the information on this interface.” and (5) “The interface was easy to view”.

After exposure to each condition, we also collected self-reported data for five usability measures: distraction, display impact on driving, ease of navigation, trust, and ease of viewing (Figure 7, Table 1). There was a significant effect of display condition on participants' reported ease of navigation, viewing, trust, and driving impact (Table 1). *Post hoc* testing showed that the screen-fixed display resulted in better usability scores for all significant differences.

#### Glance Behavior

**Figure 8:**
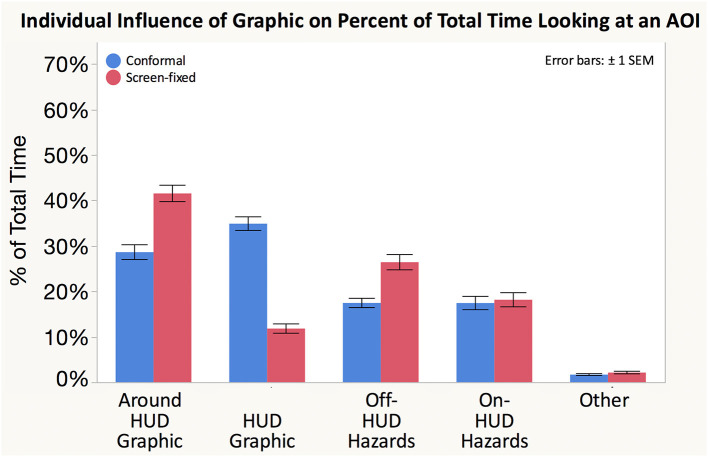
The percentage of fixations allocated to each AOI differed between Conformal and Screen-fixed displays. While there is no ideal allocation across AOIs, it is interesting to note that the percentages vary, particularly between percentage of time spent looking at and around the HUD Graphic.

We categorized areas of interest (AOIs) for participants' glance location and analyzed the AOIs two ways. The first analysis included two AOIs: on- and off-HUD. The purpose of this distinction is to understand how much drivers limit their gaze to looking only through the HUD as opposed to scanning around the scene. The second AOI coding scheme allowed us to better understand participants' scan patterns to driving-relevant areas (Figure 8). Some researchers have proposed more refined coding metrics that include locations in the roadway where hazards are likely to occur in addition to “display” and “road” glances (Seppelt et al., 2017). However, incorporating world-relative graphics into drivers' roadway scene can cause conformal HUD graphics to necessarily overlap with the road, therefore we may not be able to separate glances focused on the HUD graphic from glances focused through the HUD graphic and on the road. Therefore, this AOI coding scheme segmented the HUD into smaller AOIs, including the HUD graphic, around the HUD graphic, and on-HUD hazards. The HUD graphic included all fixations where the driver was looking directly at the graphic. However, occasionally the HUD graphic occluded the roadway ahead, and caused participants to look at locations adjacent to the HUD graphic. These glances were coded as “around HUD graphic.” When driving, around HUD glances could include regions of interest such as lane markings, hazards immediately in front of the driver. These around HUD graphic glances might also be used to resolve occlusion (e.g., make sure no hazards behind graphic). Because the HUD was positioned to afford world-fixed and world-animated graphics overlaid onto the roadway, participants may have looked through the HUD in order to check for traffic or other hazards. Thus, we coded these glances as “on-HUD hazards.” In addition to these AOIs embedded within the HUD, we also analyzed check glances toward potential cross traffic, mirrors, and other lanes. These “off-HUD hazards” encompassed all potential hazards that were visible without looking through the HUD. After tests for normality, we log transformed all eye-based response variable data though non-transformed data is shown in Figure 8.

Conformal resulted in a significantly higher maximum glance duration toward the HUD graphic than Screen-fixed. Conformal also resulted in longer mean HUD graphic glance durations than Screen-fixed. Further, the number of glances toward the HUD Graphic was significantly higher when participants used the Conformal as compared to the Screen-fixed display type. Conformal was associated with a higher percentage of time looking at the HUD Graphic than Screen-fixed. Screen-fixed resulted in a higher percentage of glances around the HUD graphic than did Conformal. There was no significant difference between the percentage of time that participants looked at off-HUD hazards, on-HUD hazards, or at the HUD in general.

In summary, because the conformal display was associated with longer average glances, higher maximum glances, higher glance count, and higher percentage of time focused on the HUD graphic specifically, participants showed a tendency to allocate more visual attention to the conformal HUD graphic than the screen-fixed graphic. Conformal was also associated with less time looking at the area around the HUD graphic and no difference in either on-HUD or off-HUD hazards, showing that the increased visual attention toward the conformal graphic did not necessarily impact participants' hazard scanning behaviors.

#### Driving Behavior

We analyzed driving data for the total duration of time in which each navigation cue (conformal arrow and screen-fixed arrow) was visible on the HUD (492 feet prior to each of the eight turns). For each turn, we calculated the relevant lateral, longitudinal, and position control metrics for each trial. We then searched each trial for times when the participant's speed was 0.0 mph and marked these as stops. For the first stop after a graphic appeared, we calculated the distance from the stopping location to the beginning of the intersection. Table 1 includes a list of the dependent driving behavioral measures, and we found no significant effects of display condition on any of the driving measures. Further, no participants missed turns at any intersections.

#### Risk-Taking

Using the Noldus video recording, we analyzed participants' risk-taking behavior by capturing how many cars out of a platoon of eight vehicles participants allowed to turn (0–8 vehicles) before deciding to make the turn themselves. If participants turned between two platoon vehicles, we also captured the gap size (in feet) of the distance between those two platoon vehicles. Data from four participants was missing due to human error and therefore we were only able to analyze the risk-taking behavior of 18 participants (out of 22). We were unable to analyze an additional five turns in conformal and two turns in screen-fixed due to simulation scenario, but the mix across turn directions was fairly even (34 L-Conformal, 33 R-Conformal, 36 L-Screen-fixed, 34 R-Screen-fixed). Display condition did not impact the number of cars that participants allowed to turn before making a turn [$X_{\left(1\right)}^{2}$ = 0.1728, *p* = 0.6776]. Of those that took a gap, there was no effect of display condition on the gap size that participants chose. Thus, the display type did not significantly impact the drivers' risk-taking behavior, nor did any participant crash into another vehicle during turns (or at any other time during the drive).

### Case Study Discussion

Our user study included 22 participants who experienced both Conformal and Screen-fixed displays while navigating in our AR DriveSim. In this study, the Screen-fixed display was associated with lower workload (measured by mental demand, effort, and overall workload) and higher usability (measured by driving demand, navigation, trust, and viewing) than the Conformal display. The difference in these self-reported measures shows that conformal AR graphics are not necessarily a inherently better user experience, and spatially locating directional graphics into the forward roadway can cause more workload in some instances.

There were no differences in driving or risk-taking behaviors despite the fact that participants using the Screen-fixed display allocated less visual attention toward the graphic and therefore, presumably allocated more visual attention toward other elements relevant to the driving task. The lack of differences in driving behaviors can be explained in a study like this because we did not include events that were unexpected or unpredictable in our driving scenarios, which might be more likely to differentiate between HUD graphics. Surprise events (unexpected or unpredictable) require rapid responses and drivers using conformal AR HUDs are especially vulnerable to change blindness or display clutter that might hinder drivers particularly in the face of unexpected events because changes in the display may mask real-world changes. Driving measures are not as sensitive as other physiological measures (Wierwille and Eggemeier, 1993) and the allocation of visual attention can be an early indicator of degraded driving ability. Thus, measures such as glance behavior provide direction about display design even when driving performance measures do not differ. Regardless of the reason for the increased visual attentional allocation, this work suggests that we should be judicious when designing AR HUDs for vehicles.

We found differences in glance behaviors with participants looking toward the Conformal display more often and for longer periods of time. It is possible that the increased visual attention that participants allocated toward the conformal display was an artifact of the study because the graphic size was bigger in the Conformal condition. However, participants may have also had to focus on the conformal graphic for a longer period of time in order to parse the navigational meaning as it scrolled in from the top of the display's field of view as participants drove forward. Thus, recent increased interest from automotive manufacturers and researchers in using conformal graphics on AR HUDs is not necessarily synonymous with safer driver behaviors and, if poorly executed, can negatively impact the user experience as well. This work indicates that in some scenarios, screen-fixed graphics may be more effective than conformal, and therefore perfectly conformal graphics may not be the solution for all AR interfaces. The temptation to incorporate realistic conformal AR graphics when designing advanced AR UIs could impede driving performance and negatively impact driver glance behaviors. However, much more work should be conducted to test expected benefits of conformal graphics when compared to other head-up UI designs. Follow-on studies should further examine how visual attention allocation toward conformal AR HUD graphics might be detrimental in instances with different road geometry, road actors, and unexpected/unpredictable events.

## AR DriveSim Discussion

The user study presented herein is an initial demonstration of how we can leverage our AR DriveSim to quickly compare UI prototypes; in this case a conformal AR hologram UI to a screen-fixed UI inspired by the same visual element (i.e., an arrow) and further examine how these UIs affect driver behavior and performance. The AR DriveSim's capabilities, however, afford *many* other types of quick exploration of AR UI designs for driving that would be otherwise by much more difficult, time consuming and/or dangerous to conduct. For example, we can examine how UI designs may move through space (e.g., animated conformal graphics) or animate on the screen, or even migrate between the road and the screen depending the context. With perfect scene geometry, vehicle tracking, and knowledge of road actors, we can examine UIs attached to other moving vehicles, pedestrians, and bicyclists without attempting to orchestrate those actors in an on-road testbed or trying to track them in real time. We can examine how much tracking error could be tolerated in an on-road AR HUD UI, or how to annotate real-world referents that are outside the AR HUD's field of view. Similarly, we can examine how to design AR UIs that can coexist in heavy traffic, where occlusion is likely to occur and creative context-aware designs need to be developed and tested. By instrumenting and actual vehicle cab with sensing devices (e.g., gesture, voice, etc.) as well as center console touch screens, we can further explore in-vehicle interaction techniques for AR in ways that would be less ideal to conduct in a completely virtual simulated driving environment with virtualized AR HUD graphics (e.g., due to challenges associated with availability of rich haptic cues typical in vehicle interfaces and rendering participants' own body in highly articulated and compelling fashion). Lastly, by using an actual optical see-through HUD (instead of simulated or virtual HUD) we can examine physiological and cognitive effects of integrating AR displays with driving scenes such as those associated with context switching and focal distance switching (Gabbard et al., 2019) which is not possible with VR-based driving simulation with simulated AR graphics. In short, AR DriveSim, is a low-cost, full-scale driving simulator with integrated AR optical see through head-up display and capabilities to quantify effects of AR UIs on driver performance and behavior. Our design provides unique and invaluable opportunities for researchers and AR HUD UI designers that cannot be met on-road or in complete VR-based simulation.

Designing, building, wiring, and programming the AR-DriveSim did not come easy, and as such, we provide a list of lessons learned on the process that may be of value to other researchers and practitioners striving to create similar cyber-physical AR testbeds (be it for driving or other AR application domains).

Regarding the physical space for a driving simulator, we recommend larger spaces over smaller ones; at least 5 × 7 m. First, a larger room affords larger cabs, which in turn support a wider range of participant sizes. Larger rooms can also better manage the excessive heat generated by the multitude of computers, displays and projectors needed. This is especially important since warm room temperatures can exacerbate simulator sickness. Larger rooms further afford placement of LCD monitors behind the cab to serve optical side mirrors and a more realistic driver experience. Taller ceilings further allow for more flexibility in purchasing and mounting projectors. If possible, ensure that the physical space contains multiple electrical circuits and a dedicated circuit to power the half cab. If the cab's interior blower fan is operational, it will be extremely useful to have the option to run the fan at its highest speed to help minimize motion sickness, although this requires significant current.

When seeking a car to use as a half cab, start by identifying cars with well-documented CAN bus IDs. This will expedite the work needed to connect the cab to the driving simulator software. Also, while it was a good idea to request that the engine and transmission be removed prior to delivery, we recommend that the Engine Control Unit remain intact to provide access to additional CAN bus data. Lastly, if CAN bus IDs are not available, do not invest much time working with simple on-board diagnostic readers, as they yield access to a subset of the total CAN bus traffic. Instead borrow or rent a formal automobile diagnostic tool from a repair shop.

Within the physical cab, we recommend routing essential cables underneath and behind trim to not only protect the cables but also to increase the quality of place illusion. That is, you want participants to believe they are in an actual driving car, not a wired-up car in a lab. Route cables for displays, IP cameras, communication, and power before completely reassembling the cab. The cab should also have adjustable seats and a robust HUD positioning and calibration process. Participants that are comfortable and have accurate view of AR content will yield higher quality data.

If possible, position the cab such that participants entering the driving simulator space enter from the driver's side. This prevents participants from having to navigate the inevitable set of cables and equipment that are present. Along these lines, we recommend that extra care be taken to manage cables by carefully choosing the right lengths and using cable management techniques. This will help minimize trip hazards for participants and experimenters.

Regarding the driving simulator software, it is our strong recommendation that researchers avoid the temptation to develop their own driving simulator software unless the software itself is the desired contribution. A complete driving simulator software solution involves much more than VR graphics including for example, the automated collection of SAE-established driving metrics, integration of real-time complex vehicle dynamics, user-friendly graphical scenario authoring tools, and so forth. While MiniSim is the option we have used, there are other commercial and open-source options available (e.g., STISIM and OpenDS).

In terms of the AR HUD software, we found that delegating the transformation tasks (e.g., MiniSim vehicle pose to X3D AR HUD pose) to Python helps simplify the experimental X3D/Javascript source code, and also helps more generally with future portability. Also, while there are likely cases where vehicle-relative coordinate system may be useful, we have found that a common absolute coordinate system greatly simplifies implementation for dynamic AR HUD graphics. This is true especially in cases where researchers do not have deep computing skills, because researchers designing scenarios can specify world-coordinates for AR HUD programmers to use on the X3D/Javascript side. Lastly, when animating conformal AR HUD graphics for turn-based navigation scenarios, we have found that single Bezier curves provide adequate definition for single-turns, and may be linked together to define more complex conditions.

## Limitations and Future Work

While there are a handful of inherent limitations of computer-based driving simulation, we present just a few limitations the driving simulator imposes on our ability to conduct AR HUD research. First, it would be difficult to conduct research related to the effects of real-world lighting and color blending on HUD usage. Even if we could luminance-match, for example, a nighttime scenario, it is not trivial to introduce glare from oncoming traffic and other lighting effects. Similarly, studying the usability of AR HUD graphics on driving backgrounds is limited by the resolution, luminance, dynamic range, and contrast of the projected driving scene. Our AR HUD simulator is also not well-positioned to study issues related to depth perception, since the fixed focal plane HUD coincidentally falls at about the same distance as the projected driving scene. We also do not yet have the ability to articulate the cab and present motion-based cues. In sum, the main limitations restrict our ability to study perceptual AR issues related to outdoor HUD usage. Such studies would need to be conducted while driving on a test track, or fixed indoors looking out.

We can easily envision near-term future work that examines the role of AR HUDs in autonomous and semi-autonomous driving. Our integration of a game controller as a secondary means to drive positions us nicely to begin this work. The testbed is also well-suited for integration of 3D spatialized audio to complement the visual HUD UIs. Lastly, we have begun to integrate gesture and voice recognition technology so that we may examine rich AR HUD interaction. Such capabilities will allow us to expand our understanding of driver distraction beyond visual attention.

## Data Availability Statement

The datasets generated for this study are available on request to the corresponding author.

## Ethics Statement

The studies involving human participants were reviewed and approved by Virginia Tech Human Research Protection Program. The patients/participants provided their written informed consent to participate in this study.

## Author Contributions

JG directed the creation and integration of all AR DriveSim components, designed the user study, and lead authoring of the manuscript. MS led the user study, conducted data analysis for user study, drafted discussion associated with user study, and edited manuscript. KT developed all software needed for the AR DriveSim tested and authored technical content related to software components. HK performed literature review, helped with logistics of setting up the AR DriveSim and edited manuscript. BJ developed all hardware/microcontroller components needed for the AR DriveSim tested, authored technical content related to software components.

### Conflict of Interest

The authors declare that the research was conducted in the absence of any commercial or financial relationships that could be construed as a potential conflict of interest.
